# Development and preliminary evaluation of a Neuman Systems Model–based protocol for postoperative delirium prevention in patients undergoing pancreatic cancer surgery

**DOI:** 10.1097/MD.0000000000050172

**Published:** 2026-08-21

**Authors:** Shujia Wang, Li Yu, Wenli Xu

**Affiliations:** a Department of General Surgery, Ruijin Hospital, School of Medicine, Shanghai Jiao Tong University, Shanghai City, China; b Nursing Ward, Ruijin Hospital, School of Medicine, Shanghai Jiao Tong University, Shanghai City, China.

**Keywords:** Neuman model, pancreatic cancer, postoperative delirium, prevention and management protocol, quasi-experimental study

## Abstract

This study aimed to develop and evaluate a Neuman Systems Model–based protocol for the prevention and management of postoperative delirium in patients undergoing surgery for pancreatic cancer. The protocol was developed through literature review and a modified Delphi process, and its clinical effectiveness was assessed in a single-center study using a historical control design. A total of 120 patients who underwent radical resection for pancreatic cancer between January 2024 and December 2025 were included, with 60 patients in the control group and 60 in the intervention group. Compared with the control group, the intervention group had a significantly lower incidence of postoperative delirium (10.0% vs 26.7%, *P* = .018), lower peak Confusion Assessment Method-S scores, shorter delirium duration, and a lower overall incidence of delirium-related adverse events. The intervention group also showed better quality of life scores at 1 and 3 months after surgery, lower depression, anxiety, and stress scores at postoperative day 7, better postoperative recovery indicators, and higher nursing satisfaction. Supplementary analyses identified age ≥70 years, preoperative sleep disturbance, postoperative electrolyte imbalance, postoperative infection, and preoperative risk of malnutrition as independent factors associated with postoperative delirium. These findings suggest that the Neuman Systems Model–based protocol may help reduce the incidence and severity of postoperative delirium and improve selected postoperative outcomes in patients with pancreatic cancer, although further validation in larger multicenter studies is needed.

## 1. Introduction

Pancreatic cancer is among the most aggressive malignancies of the digestive system, featuring an insidious onset, rapid disease progression, poor prognosis, and high mortality. According to the Global Burden of Disease study, the numbers of deaths, incident cases, and disability-adjusted life years attributable to pancreatic cancer increased steadily from 1990 to 2017, highlighting its growing impact as a major global public health concern.^[1]^ Recent cancer statistics from the United States further confirm that pancreatic cancer remains one of the leading causes of cancer-related death, with its disease burden continuing to rise.^[2]^ For patients who are candidates for surgery, radical resection remains the cornerstone of treatment and offers the best chance for prolonged survival. Nevertheless, pancreatic surgery is typically complex and highly invasive, with a substantial risk of perioperative complications. After surgery, patients commonly face a range of physiological and psychological challenges, including pain, sleep disruption, infection, malnutrition, glycemic instability, and fluid-electrolyte imbalance. These cumulative stressors can delay postoperative recovery and may further increase the likelihood of postoperative delirium.^[3]^

Postoperative delirium (POD) is a common neuropsychiatric complication during the perioperative period and is typically characterized by acute onset, fluctuating course, inattention, disturbed consciousness, cognitive dysfunction, and disorganized thinking.^[4]^ Accumulating evidence indicates that POD is associated with prolonged hospital stay, increased healthcare expenditure, delayed functional recovery, a higher risk of adverse events, and poor long-term prognosis, thereby posing a substantial challenge to perioperative management.^[5]^ Given the multifactorial pathogenesis of delirium, single preventive measures have shown limited effectiveness. In contrast, multicomponent non-pharmacological interventions targeting modifiable risk factors are currently regarded as one of the most effective evidence-based strategies for delirium prevention. A meta-analysis by Hshieh et al demonstrated that such interventions can significantly reduce the incidence of delirium and falls and may also contribute to shorter hospitalization.^[5]^ Consistently, the evidence-based and consensus guidelines issued by the European Society of Anaesthesiologists emphasize that early identification of high-risk patients, perioperative optimization, continuous postoperative surveillance, and multidisciplinary collaboration are essential for the prevention and management of POD.^[6]^

Postoperative delirium is a well-recognized complication in pancreatic surgery. Gallagher et al found that the incidence of delirium following pancreaticoduodenectomy was about 14%, and that affected patients were more likely to experience a complicated postoperative course and an increased risk of complications; advanced age was identified as an independent predictor.^[7]^ Consistent with these findings, Ito et al also reported that postoperative delirium is common after pancreaticoduodenectomy and is associated with distinct clinical risk factors.^[8]^ Recent evidence further indicates that the development of delirium after pancreatic cancer surgery is closely associated with advanced age, higher American Society of Anesthesiologists grade, perioperative infection, electrolyte disorders, malnutrition, and drug exposure, and that it occurs predominantly in the early postoperative phase.^[9,10]^ Collectively, these findings suggest that delirium after pancreatic cancer surgery shares the core clinical features of POD while also being influenced by the unique perioperative characteristics of pancreatic procedures, including extensive surgical trauma, substantial metabolic instability, and a high burden of postoperative complications. As a result, routine delirium prevention measures may not be sufficient for this high-risk population, underscoring the need for more targeted and integrated preventive strategies.

Although substantial progress has been made in identifying risk factors for postoperative delirium and developing non-pharmacological preventive strategies, the existing evidence is derived mainly from geriatric medical wards, general surgical settings, or broader perioperative populations. By contrast, preventive management strategies specifically designed for patients undergoing pancreatic cancer surgery remain insufficiently investigated. Most available studies have focused on retrospective analyses of risk factors or the evaluation of isolated nursing measures, with relatively few proposing an integrated management model grounded in a clear theoretical framework that incorporates preoperative assessment, perioperative surveillance, environmental regulation, family participation, psychological support, and continuity of care into a unified pathway.^[6-10]^ Notably, patients recovering from pancreatic cancer surgery are exposed not only to substantial physiological stressors, such as pain, indwelling catheter management, infection, and nutritional or metabolic disturbances, but also to pronounced psychological distress related to disease prognosis, postoperative functional recovery, and changes in family and social roles. Therefore, the prevention and management of postoperative delirium in this population should not be regarded solely as an issue of symptom identification, but rather as a complex clinical challenge arising from the interaction of physiological, psychological, environmental, and social factors.

The Neuman Systems Model provides a particularly valuable theoretical framework for understanding this issue. It conceptualizes the individual as an open system in constant interaction with the internal and external environment, emphasizing that intrapersonal, interpersonal, and extrapersonal stressors jointly affect system stability. The model further advocates early risk identification, timely intervention, and functional restoration through primary, secondary, and tertiary prevention. A review by Skalski et al based on the Neuman Systems Model suggested that the concept of stressors enables nurses to classify risk factors in complex clinical situations in a more structured manner and offers a systematic basis for intervention planning.^[11]^ In the perioperative setting, a randomized study by Akhlaghi et al showed that interventions guided by the Neuman Systems Model could reduce anxiety and stress in patients undergoing coronary artery bypass grafting and improve perioperative psychological adaptation.^[12,13]^ Collectively, these findings indicate that the Neuman Systems Model has particular strengths in addressing clinical problems that are multifactorial, multidimensional, and dynamically evolving, and is especially well suited for integrating physiological, psychological, and environmental management in patients undergoing major surgery. For postoperative delirium in patients with pancreatic cancer, a condition characterized by high risk, clinical complexity, and potential preventability, a continuous prevention and management strategy guided by the Neuman Systems Model and targeting multiple domains, including preoperative risk status, postoperative physiological stress, environmental stimulation, and family support, may facilitate earlier identification, stratified intervention, and sustained management of delirium.

Accordingly, the present study adopts the Neuman Systems Model as its guiding theoretical framework. Through a systematic review of risk factors for postoperative delirium in patients with pancreatic cancer, together with the available nursing evidence, this study seeks to develop and clinically validate a targeted delirium prevention and management protocol for this population. By integrating preoperative risk assessment, perioperative physiological stress management, environmental optimization, psychological support, family participation, and continuity of care, the study is expected to provide both a theoretical foundation and a practical reference for the prevention and management of postoperative delirium in pancreatic cancer patients. It may also offer supportive evidence for improving perioperative outcomes and enhancing the quality of nursing care.

## 2. Materials and methods

### 2.1. Study design

This study was approved by the Ethics Committee of Ruijin Hospital, School of Medicine, Shanghai Jiao Tong University (approval no.2025-SHJTU-kf1219). The study was conducted in 2 phases. The first phase focused on protocol development. Guided by the Neuman Systems Model, a prevention and management protocol for postoperative delirium in patients with pancreatic cancer was formulated through a literature review combined with a modified Delphi expert consultation process. The second phase involved preliminary clinical evaluation. Using a historical control design within a quasi-experimental framework, this phase compared patient outcomes before and after the implementation of the Neuman Systems Model–based postoperative delirium prevention and management program.

### 2.2. Theoretical framework

The Neuman Systems Model conceptualizes the individual as an open system that is continuously exposed to multidimensional stressors, including physiological, psychological, sociocultural, developmental, and spiritual factors. Within this framework, nursing interventions are directed toward identifying and alleviating these stressors in order to preserve system stability through primary, secondary, and tertiary prevention.^[11-13]^ In the present study, the Neuman Systems Model was adopted as the guiding theoretical framework. Based on physiological, psychological, environmental, and social support factors associated with postoperative delirium in patients with pancreatic cancer, a prevention and management program was constructed to span the preoperative, early postoperative, and recovery stages. This framework provides a theoretical basis for the early identification, stratified intervention, and continuous management of postoperative delirium in this patient population.

### 2.3. Development of a prevention and management protocol

#### 2.3.1. Literature review

The evidence search used to develop the clinical protocol was completed before the implementation of the intervention and covered each database from inception to November 30, 2024. The intervention protocol was subsequently finalized before its clinical implementation in January 2025. An updated search was conducted through March 31, 2026, solely to revise the background and discussion of the manuscript. Evidence identified during the updated search did not retrospectively alter the intervention delivered to the study participants.

The databases searched included PubMed/MEDLINE, Embase, Web of Science, the Cochrane Library, CNKI, Wanfang Data, and VIP. Controlled vocabulary and free-text terms related to pancreatic cancer or pancreatic surgery, postoperative delirium, perioperative prevention and management, multicomponent non-pharmacological interventions, nursing care, and the Neuman Systems Model were combined. Search terms were adapted to the syntax of each database, and no language restriction was applied during the initial search.

The PubMed search strategy was as follows: ((“Pancreatic Neoplasms”[Mesh] OR pancreatic cancer*[Title/Abstract] OR pancreatic neoplasm*[Title/Abstract] OR pancreatectom*[Title/Abstract] OR pancreaticoduodenectom*[Title/Abstract]) AND (“Delirium”[Mesh] OR delirium[Title/Abstract] OR postoperative delirium[Title/Abstract]) AND (prevent*[Title/Abstract] OR management[Title/Abstract] OR intervention*[Title/Abstract] OR nursing[Title/Abstract] OR perioperative care[Title/Abstract])) OR ((“Neuman Systems Model”[Title/Abstract] OR “Neuman System Model”[Title/Abstract]) AND (nursing[Title/Abstract] OR perioperative[Title/Abstract] OR surgery[Title/Abstract])).

Eligible publications included clinical guidelines, systematic reviews, controlled studies, cohort studies, and relevant evidence summaries addressing postoperative delirium after pancreatic or major abdominal surgery, multicomponent delirium prevention, or application of the Neuman Systems Model. Studies involving only pediatric populations, animal experiments, alcohol-withdrawal delirium, or postoperative cognitive dysfunction without delirium outcomes were excluded.

#### 2.3.2. Modified Delphi expert consultation

Based on the literature review, the draft protocol was revised and refined using a modified Delphi approach. Experts were eligible for inclusion if they met the following criteria: involvement in pancreatic surgery, nursing, critical care, nutritional support, psychological care, or postoperative delirium management; substantial clinical or research experience; familiarity with the Neuman Systems Model or with protocols for postoperative delirium management; and willingness to participate in the study and provide timely feedback. A total of 15 to 20 experts were invited, representing the fields of pancreatic surgery, nursing, critical care, psychological support, and nutritional management.

Two rounds of expert consultation were conducted using electronic questionnaires. The first round focused primarily on evaluating the overall framework of the protocol, the primary and secondary indicators, and specific items, while also soliciting suggestions for additions, deletions, and revisions. The second round was conducted mainly to reevaluate the importance and feasibility of the revised items. All items were scored using a 5-point Likert scale. The consultation results were assessed using the expert consensus coefficient, authority coefficient, mean item score, coefficient of variation, and Kendall’s coefficient of concordance. In principle, items with a mean score of at least 3.5 and a coefficient of variation below 0.25 were retained. For items with values close to these thresholds but considered clinically important, the research team made the final decision after discussion. Based on the findings from the 2 consultation rounds, a Neuman Systems Model–based protocol for the prevention and management of postoperative delirium in patients with pancreatic cancer was finalized.

#### 2.3.3. Content of the prevention and management protocol

The final prevention and management protocol comprised the following components:

1. Establishment of a multidisciplinary management team: the team consisted of pancreatic surgeons, ICU physicians and nurses, specialist nurses, dietitians, and psychological support staff. All members implemented the protocol after receiving standardized training on the study procedures.
2. Preoperative risk assessment and health education: within 24 hours of admission, the responsible nurse completed screening for high-risk factors for postoperative delirium, sleep assessment, nutritional risk assessment, and an initial psychological evaluation, and provided targeted health education to patients and their family members.
3. Preoperative psychological support and targeted intervention: a specialist nurse provided daily psychological counseling sessions lasting approximately 15 to 20 minutes to help patients understand the surgical procedure, common postoperative symptoms, and key measures for delirium prevention, thereby reducing anxiety and uncertainty.
4. Perioperative physiological stress management: continuous management was applied to key domains, including pain, sleep, fluid and electrolyte balance, infection, nutrition, and blood glucose. Vital signs, laboratory parameters, and sleep status were monitored daily after surgery, with intensified observation for high-risk patients.
5. Environmental optimization and orientation support: patients’ circadian rhythm and orientation were maintained through measures such as reducing nighttime light and noise, minimizing unnecessary disturbances, providing clocks and calendars, and strengthening orientation-based communication by healthcare staff.
6. Family involvement and support: family members were instructed to participate in orientation support and bedside companionship twice daily, with each session lasting 10 to 15 minutes, in order to enhance the patient’s emotional stability and sense of reality.
7. Dynamic delirium assessment and stratified intervention: nurses who had received standardized training conducted assessments twice daily, at 8:00 am and 8:00 pm, beginning once the patient regained consciousness after surgery. For high-risk patients or those with abnormal manifestations, assessment frequency was increased, and individualized management measures were implemented according to the established protocol.
8. Continuity of care: rehabilitation guidance was provided before discharge, and follow-up assessments were conducted at 1 month and 3 months after surgery to evaluate quality of life, emotional status, and recovery progress.

These measures covered the entire continuum from the preoperative period to the early postoperative and recovery phases, reflecting an integrated management approach based on primary, secondary, and tertiary prevention.

#### 2.3.4. Operational mapping of the Neuman Systems Model to protocol components

Each protocol component was aligned with the Neuman Systems Model according to the main stressor involved, the level of prevention, the intended system response, and the corresponding clinical action. Preoperative risk assessment addressed intrapersonal stressors such as advanced age, sleep disturbance, malnutrition, anxiety, and physiological vulnerability. As a primary prevention strategy, it aimed to strengthen the patient’s defenses through early screening, individualized assessment, and timely correction of modifiable risks. Health education and psychological support targeted intrapersonal and interpersonal stressors related to uncertainty, fear, and insufficient knowledge. These primary prevention measures were intended to improve psychological stability, coping ability, and treatment cooperation. Perioperative physiological management focused on pain, infection, electrolyte imbalance, glycemic fluctuation, malnutrition, and sleep disruption. It combined primary prevention, by reducing exposure to these stressors, with secondary prevention, by identifying and correcting abnormalities at an early stage. Environmental optimization and orientation support addressed extrapersonal stressors such as noise, excessive light, frequent interruptions, and unfamiliar surroundings. These measures aimed to maintain circadian rhythm, orientation, and cognitive stability through noise reduction, sleep protection, clocks, calendars, and repeated orientation cues. Family involvement mainly addressed interpersonal stressors and provided emotional reassurance, familiar communication, and orientation support. It contributed primarily to primary prevention and also supported patients during delirium management and recovery. Dynamic delirium assessment and stratified intervention represented secondary prevention. Regular the Confusion Assessment Method (CAM) and CAM-S assessments enabled early identification of cognitive or behavioral changes, followed by prompt investigation of possible causes and individualized management. Rehabilitation guidance and follow-up represented tertiary prevention. These measures aimed to restore system stability, promote functional recovery, and support long-term adaptation through discharge education, rehabilitation planning, and follow-up at 1 and 3 months.

Overall, the protocol translated the Neuman Systems Model into a practical pathway in which primary prevention reduced exposure to stressors, secondary prevention supported early detection and treatment, and tertiary prevention promoted recovery and continued adaptation.

### 2.4. Validation of the prevention and management protocol

#### 2.4.1. Study population and grouping

Patients undergoing curative surgery for pancreatic cancer at Ruijin Hospital were identified using identical eligibility criteria for both study periods. The historical control group comprised consecutive eligible patients treated from January 1, 2024, to December 31, 2024, before the implementation of the protocol. The intervention group comprised consecutive eligible patients treated from January 1, 2025, to December 31, 2025, after the completion of protocol development and staff training. Both groups were managed in the same specialized ward by a relatively stable multidisciplinary team. The same inclusion and exclusion criteria, outcome definitions, and postoperative observation window were applied to both groups.

For the historical control group, medical records were reviewed using a standardized data-extraction form. Of the potentially eligible historical records screened, records that did not meet the eligibility criteria or contained insufficient data for the primary outcome assessment were excluded. The final analysis included 60 patients in the historical control group and 60 patients in the intervention group. Apart from the study intervention, routine perioperative clinical and nursing practices were maintained as consistently as possible across the 2 periods. Nevertheless, residual temporal confounding inherent in the historical control design could not be completely excluded.

#### 2.4.2. Inclusion criteria

1. Histopathological or cytological confirmation of pancreatic cancer;
2. Undergoing curative resection of the pancreatic tumor;
3. Age ≥ 18 years;
4. Clear consciousness and intact orientation before surgery, with no evidence of delirium and the ability to cooperate with relevant assessments;
5. Provision of informed consent for study participation.

#### 2.4.3. Exclusion criteria

1. History of cognitive impairment, dementia, or other severe neurological disorders;
2. History of severe psychiatric illness or inability to cooperate with the assessments;
3. Significant alcohol or substance dependence;
4. Severe systemic disease, such as advanced cardiac, pulmonary, hepatic, or renal failure, likely to substantially affect perioperative recovery;
5. History of postoperative delirium or presence of severe preoperative conditions, such as marked hypoxemia or electrolyte disturbance, that could substantially interfere with delirium assessment.

#### 2.4.4. Sample size estimation

The incidence of postoperative delirium was used as the primary outcome measure in this study. Based on findings from previous studies on perioperative delirium and the results of a pilot investigation, the incidence of postoperative delirium was estimated to be approximately 25% in the control group and was expected to decrease to 10% in the intervention group. With a significance level of α = 0.05 and a statistical power of 1 − β = 0.80, the sample size calculated using the formula for comparing 2 independent proportions indicated that approximately 50 patients were required in each group. After accounting for a potential dropout or incomplete data rate of 20%, the final sample size was determined to be 60 patients per group, yielding a total sample of 120 participants.

#### 2.4.5. Control group intervention

The control group received standard perioperative care together with routine delirium prevention measures, including preoperative assessment, psychological support and health education, basic nutritional support, fluid and electrolyte management, intraoperative vital sign monitoring, postoperative pain control, maintenance of the sleep–wake cycle, surveillance for postoperative complications, and routine identification and management of delirium.

#### 2.4.6. Intervention in the experimental group

In addition to standard perioperative care, the intervention group received a Neuman Systems Model–based protocol for the prevention and management of postoperative delirium in patients with pancreatic cancer. The protocol was delivered by a multidisciplinary team that had received standardized training and included the following components:

1. Completion of high-risk factor screening, sleep assessment, nutritional risk assessment, and an initial psychological evaluation within 24 hours of admission;
2. Provision of targeted health education and daily psychological support before surgery;
3. Daily dynamic monitoring of postoperative pain, fluid and electrolyte balance, infection, blood glucose, and nutritional status;
4. Reduction of adverse environmental stimuli through environmental optimization, maintenance of circadian rhythms, and orientation support;
5. Involvement of family members in orientation assistance and emotional reassurance;
6. Ongoing assessment using standardized delirium assessment tools, with timely initiation of stratified management for high-risk patients;
7. Provision of rehabilitation education before discharge, together with follow-up at 1 month and 3 months after surgery.

To ensure consistency of implementation, all study personnel received standardized training before the study commenced, and standardized management checklists were used to document adherence to each component of the protocol.

### 2.5. Observation indicators and definitions

#### 2.5.1. Primary outcomes

Incidence of postoperative delirium: the Chinese version of the CAM was used to identify postoperative delirium. Screening began after recovery of consciousness and was performed twice daily, at 08:00 and 20:00, until postoperative day 7 or discharge, whichever occurred first. Additional assessments were performed whenever delirium-like cognitive or behavioral changes were observed. All assessors received standardized training in the administration of the CAM and CAM-S and were required to pass a competency assessment before participating in data collection. Assessments were conducted by clinical nurses involved in patient care; therefore, independence from intervention delivery and blinding to group allocation could not be ensured. This lack of assessor independence and blinding may have introduced detection bias, particularly for behavioral and cognitive outcomes, and was therefore acknowledged as a limitation of the study. The incidence of postoperative delirium was defined as the proportion of analyzed patients who met the CAM diagnostic criteria at least once during the observation period.^[14]^

#### 2.5.2. Secondary outcomes

1. Severity and duration of delirium: delirium severity was evaluated using the short-form Confusion Assessment Method Severity scale (CAM-S), with total scores ranging from 0 to 7; higher scores indicate greater delirium severity. Delirium duration was defined as the number of days from the initial onset of delirium until consecutive assessments indicated a return to normal status.^[15]^
2. Delirium-related adverse events: these included unplanned extubation, skin injury, falls, and bed falls.
3. Quality of life: the 36-Item Short Form Health Survey (SF-36) was used to assess physical functioning, social functioning, mental health, and overall quality of life at 1 month and 3 months after surgery. Higher scores indicated better quality of life.
4. Negative emotions: the Chinese version of the Depression Anxiety Stress Scales–21 (DASS-21) was used to assess levels of depression, anxiety, and stress before surgery and on postoperative day 7. Higher scores indicated greater emotional distress.
5. Postoperative recovery indicators: these included the length of hospital stay, total postoperative drainage volume, incidence of pancreatic fistula, incidence of infection, mean postoperative blood glucose level, and postoperative serum albumin level.
6. Nursing satisfaction: A self-developed nursing satisfaction questionnaire was used to evaluate patients’ satisfaction with nursing care. Responses were categorized as “very satisfied,” “satisfied,” “neutral,” or “dissatisfied,” and the overall satisfaction rate was calculated.

#### 2.5.3. Supplementary analysis variables

To further examine factors associated with postoperative delirium, the following variables were additionally collected: age ≥ 70 years, preoperative sleep disturbance, electrolyte imbalance within 7 days after surgery, infection within 7 days after surgery, preoperative risk of malnutrition, pain score at 24 hours postoperatively, and preoperative anxiety score as measured by the DASS-21. Electrolyte imbalance within 7 days postoperatively was defined as any abnormal sodium, potassium, or calcium level detected during the first 7 postoperative days. Infection within 7 days postoperatively was defined as any clinically diagnosed infection occurring during the same period. Preoperative risk of malnutrition was determined according to the results of nutritional screening.^[16]^

### 2.6. Data collection and quality control

This single-center retrospective cohort study reviewed patients who underwent curative surgery for pancreatic cancer between January 1, 2024, and December 31, 2025. The control group included patients treated in 2024 before protocol implementation, whereas the intervention group included patients treated in 2025 after the implementation of the Neuman Systems Model–based protocol. Data for both groups were retrospectively extracted from electronic medical records, nursing records, laboratory systems, assessment forms, and follow-up records using a standardized data-extraction form. The same variable definitions, outcome criteria, and observation periods were applied to both groups. Baseline and perioperative variables were collected from admission until postoperative day 7 or discharge, whichever occurred first. CAM and CAM-S assessments were documented twice daily during this period. DASS-21 scores were recorded preoperatively and on postoperative day 7, SF-36 scores at 1 and 3 months after surgery, and nursing satisfaction before discharge. Two researchers independently extracted and cross-checked the data. Discrepancies were resolved by reviewing the original records. Missing data were documented by variable and group, and complete-case analysis was used for the corresponding outcomes. Protocol adherence in the intervention group was assessed from completed checklists and nursing documentation.

### 2.7. Statistical methods

Data were analyzed using IBM SPSS version 27.0 (IBM Corp). Continuous variables were first assessed for normality. Variables with a normal distribution were expressed as mean ± standard deviation (*x̄* ± *s*), and between-group comparisons were performed using the independent-samples *t* test. Variables that did not follow a normal distribution were analyzed using appropriate nonparametric tests. Categorical variables were presented as frequencies and percentages, and comparisons between groups were conducted using the chi-square test or Fisher’s exact test.

For outcomes measured at multiple time points, such as quality of life and negative emotions, between-group comparisons were performed separately at each time point. Postoperative delirium was used as the dependent variable, and variables showing statistical significance in univariate analyses were entered into a multivariable logistic regression model. Stepwise regression was applied to identify factors associated with postoperative delirium, and odds ratios (ORs) with 95% confidence intervals (CIs) were reported. All tests were 2-sided, and a *P* value < .05 was considered statistically significant.

### 2.8. Ethical considerations

This study was reviewed and approved by the hospital’s Institutional Review Board. All participants were fully informed about the study and provided written informed consent prior to enrollment. Throughout the study, patient privacy and data confidentiality were strictly protected, and all collected data were used exclusively for research purposes.

## 3. Results

### 3.1. Patient enrollment and comparison of general characteristics between the 2 groups

A total of 120 eligible patients who underwent surgery for pancreatic cancer were included in this study. All participants were successfully allocated to the study groups and completed outcome assessment, with 60 patients in the intervention group and 60 in the control group. Comparisons of baseline characteristics between the 2 groups, including age, sex, body mass index, educational level, surgical approach, tumor differentiation grade, comorbidities, and American Society of Anesthesiologists classification, showed no statistically significant differences (all *P* > .05), indicating good baseline comparability. Details are presented in Table 1.

**Table 1:** Comparison of general characteristics between the 2 groups.

Variable	Intervention group (n = 60)	Control group (n = 60)	*t*/χ^2^ value	*P* value
Age (yr)	63.8 ± 8.7	64.6 ± 9.1	0.492	.623
Sex (male/female)	35/25	36/24	0.034	.853
BMI (kg/m^2^)	22.4 ± 3.1	22.1 ± 3.4	0.505	.614
Educational level (high school or below/college or above)	26/34	24/36	0.137	.711
Surgical approach (pancreaticoduodenectomy/distal pancreatectomy)	39/21	41/19	0.150	.699
Tumor differentiation grade (high/moderate/low)	17/30/13	18/29/13	0.046	.977
Comorbidities (yes/no)	29/31	31/29	0.133	.715
ASA classification (I–II/III)	38/22	36/24	0.141	.707

ASA = American Society of Anesthesiologists.

### 3.2. Comparison of postoperative delirium incidence between the 2 groups

In the intervention group, 6 patients developed postoperative delirium, corresponding to an incidence of 10.0%, whereas 16 patients in the control group developed postoperative delirium, corresponding to an incidence of 26.7%. The between-group difference was statistically significant (χ^2^ = 5.566, *P* = .018). Compared with the control group, the intervention group showed an absolute reduction of 16.7 percentage points in the incidence of postoperative delirium.

With respect to delirium severity, the peak CAM-S score was significantly lower in the intervention group than in the control group (*t* = 3.089, *P* = .003). Delirium duration was also significantly shorter in the intervention group than in the control group (*t* = 3.552, *P* = .003). Detailed results are presented in Table 2.

**Table 2:** Comparison of postoperative delirium incidence between the 2 groups.

Variable	Intervention group (n = 60)	Control group (n = 60)	*t*/χ^2^ value	*P* value
Number of patients with delirium	6	16	5.566	.018
Incidence of delirium (%)†	10.0	26.7		
Peak CAM-S score	0.82 ± 1.46	1.93 ± 2.37	3.089	.003
Duration of delirium (days)*	2.3 ± 0.8	4.5 ± 1.6	3.552	.003

* Delirium duration was calculated only among patients who developed delirium (intervention group, n = 6; control group, n = 16).

† Between-group comparisons of delirium incidence were performed using the chi-square test.

### 3.3. Comparison of delirium-related adverse events between the 2 groups

In the intervention group, the incidences of unplanned extubation, skin injury, and falls/bed falls were 1.7%, 1.7%, and 0.0%, respectively, compared with 6.7%, 5.0%, and 3.3% in the control group. No statistically significant between-group differences were observed for any individual adverse event (all *P* > .05). However, the overall incidence of delirium-related adverse events was 3.3% in the intervention group and 13.3% in the control group, and this difference was statistically significant (χ^2^ = 3.927, *P* = .048). Detailed results are shown in Table 3.

**Table 3:** Comparison of delirium-related adverse events between the 2 groups.

Variable	Intervention group (n = 60)	Control group (n = 60)	χ^2^ value	*P* value
Unplanned extubation	1 (1.7)	4 (6.7)	1.878	.171
Skin injury	1 (1.7)	3 (5.0)	1.034	.309
Falls/bed falls	0 (0.0)	2 (3.3)	2.034	.154
Overall incidence	2 (3.3)	8 (13.3)	3.927	.048

The overall incidence was calculated based on the number of patients who experienced at least 1 delirium-related adverse event; patients with multiple events were counted only once.

### 3.4. Comparison of quality of life between the 2 groups

At 1 month after surgery, the intervention group had significantly higher SF-36 scores than the control group in physical functioning, social functioning, mental health, and total score (all *P* < .05). At 3 months postoperatively, the intervention group continued to show significantly higher scores than the control group across these same dimensions (all *P* < .05). Detailed results are presented in Table 4.

**Table 4:** Comparison of quality of life between the 2 groups.

Variable	Time point	Intervention group	Control group	*T* value	*P* value
Physical functioning	1 mo postoperatively	68.5 ± 10.2	61.4 ± 11.3	3.619	<.001
	3 mo postoperatively	79.6 ± 9.4	72.2 ± 10.1	4.143	<.001
Social functioning	1 mo postoperatively	63.1 ± 9.6	57.3 ± 10.7	3.116	.002
	3 mo postoperatively	74.8 ± 8.8	68.0 ± 9.6	4.065	<.001
Mental health	1 mo postoperatively	60.4 ± 8.7	54.1 ± 9.2	3.857	<.001
	3 mo postoperatively	72.6 ± 8.1	65.8 ± 8.7	4.412	<.001
Total score	1 mo postoperatively	61.2 ± 8.6	55.0 ± 9.4	3.769	<.001
	3 mo postoperatively	72.8 ± 7.9	66.4 ± 8.5	4.272	<.001

The data at each time point represent the results of between-group comparisons.

### 3.5. Comparison of negative emotions between the 2 groups

Before surgery, no statistically significant differences were observed between the 2 groups in DASS-21 scores for depression, anxiety, or stress (all *P* > .05). By postoperative day 7, the intervention group had significantly lower scores for depression, anxiety, and stress than the control group (all *P* < .001). Detailed results are presented in Table 5.

**Table 5:** Comparison of negative emotions between the 2 groups.

Variable	Time point	Intervention group	Control group	*T* value	*P* value
Depression	Preoperative	7.1 ± 2.8	7.4 ± 2.9	0.579	.564
	Postoperative day 7	5.2 ± 2.4	7.0 ± 2.8	3.777	<.001
Anxiety	Preoperative	6.8 ± 2.7	7.0 ± 2.8	0.396	.693
	Postoperative day 7	4.8 ± 2.1	6.6 ± 2.6	4.178	<.001
Stress	Preoperative	8.3 ± 3.1	8.5 ± 3.0	0.358	.721
	Postoperative day 7	5.9 ± 2.5	8.1 ± 2.9	4.442	<.001

The data at each time point represent the results of between-group comparisons.

### 3.6. Comparison of postoperative recovery indicators between the 2 groups

Compared with the control group, the intervention group had a shorter postoperative length of stay, lower total postoperative drainage volume, lower mean postoperative blood glucose levels, and higher postoperative albumin levels, with all differences reaching statistical significance (all *P* < .05). Although the incidences of pancreatic fistula and infection were lower in the intervention group than in the control group, these differences were not statistically significant (all *P* > .05). Detailed results are presented in Table 6.

**Table 6:** Comparison of postoperative recovery indicators between the 2 groups.

Variable	Intervention group	Control group	*t*/χ^2^ value	*P* value
Postoperative length of stay (d)	15.6 ± 4.3	19.1 ± 5.7	3.797	<.001
Total postoperative drainage volume (mL)	462 ± 118	538 ± 136	3.270	.001
Incidence of pancreatic fistula	7 (11.7)	11 (18.3)	1.046	.306
Incidence of infection	5 (8.3)	12 (20.0)	3.358	.067
Mean postoperative blood glucose (mmol/L)	8.2 ± 1.4	9.0 ± 1.8	2.717	.008
Postoperative albumin (g/L)	33.8 ± 4.1	31.5 ± 4.3	2.999	.003

### 3.7. Comparison of patient satisfaction between the 2 groups

The overall nursing satisfaction rate was 96.7% in the intervention group and 83.3% in the control group, with a statistically significant difference between the 2 groups (χ^2^ = 5.926, *P* = .015). Detailed results are presented in Table 7.

**Table 7:** Comparison of nursing satisfaction between the 2 groups.

Variable	Intervention group (n = 60)	Control group (n = 60)	χ^2^ value	*P* value
Very satisfied	34 (56.7)	24 (40.0)		
Satisfied	24 (40.0)	26 (43.3)		
Neutral	2 (3.3)	8 (13.3)		
Dissatisfied	0 (0.0)	2 (3.3)		
Overall satisfaction rate	58 (96.7)	50 (83.3)	5.926	.015

### 3.8. Supplementary analysis: factors associated with postoperative delirium

The 120 patients were categorized into a delirium group (n = 22) and a non-delirium group (n = 98) according to the occurrence of postoperative delirium. Univariate analysis showed that age ≥ 70 years, preoperative sleep disturbance, electrolyte imbalance within 7 days after surgery, infection within 7 days after surgery, preoperative risk of malnutrition, pain score within 24 hours postoperatively, and preoperative anxiety score (DASS-21) were all significantly associated with postoperative delirium (all *P* < .05). Detailed results are presented in Table 8.

**Table 8:** Univariate analysis of factors associated with postoperative delirium.

Variable	Delirium group (n = 22)	Non-delirium group (n = 98)	*t*/χ^2^ value	*P* value
Age ≥ 70 years, n (%)	14 (63.6)	28 (28.6)	9.710	.002
Preoperative sleep disturbance, n (%)	13 (59.1)	24 (24.5)	10.086	.001
Electrolyte imbalance within 7 d postoperatively, n (%)	12 (54.5)	20 (20.4)	10.707	.001
Infection within 7 d postoperatively, n (%)	10 (45.5)	17 (17.3)	8.140	.004
Preoperative risk of malnutrition, n (%)	11 (50.0)	18 (18.4)	9.810	.002
Postoperative pain score (24 h)	5.8 ± 1.4	4.3 ± 1.2	4.656	<.001
Preoperative anxiety score (DASS-21)	9.4 ± 3.1	6.6 ± 2.8	3.895	<.001

Variables that were statistically significant in the univariate analysis were subsequently entered into a multivariable logistic regression model. The results showed that age ≥ 70 years, preoperative sleep disturbance, electrolyte imbalance within 7 days postoperatively, infection within 7 days postoperatively, and preoperative risk of malnutrition were independently associated with postoperative delirium (all *P* < .05). Among these factors, electrolyte imbalance within 7 days after surgery (OR = 3.63, 95% CI: 1.34–9.83) and age ≥ 70 years (OR = 3.46, 95% CI: 1.25–9.58) demonstrated relatively stronger associations with postoperative delirium. Detailed results are presented in Table 9.

**Table 9:** Multivariable logistic regression analysis of factors associated with postoperative delirium.

Variable	β	SE	Wald χ^2^	OR	95% CI	*P* value
Age ≥ 70 yr	1.24	0.52	5.69	3.46	1.25–9.58	.017
Preoperative sleep disturbance	1.10	0.49	5.04	3.00	1.15–7.82	.025
Electrolyte imbalance within 7 d postoperatively	1.29	0.51	6.40	3.63	1.34–9.83	.011
Infection within 7 d postoperatively	1.03	0.50	4.24	2.80	1.04–7.52	.040
Preoperative risk of malnutrition	0.99	0.48	4.25	2.69	1.05–6.92	.039

CI = confidence interval, OR = odds ratio, SE = standard error.

## 4. Discussion

This study developed a Neuman Systems Model–based protocol for the prevention and management of postoperative delirium in patients with pancreatic cancer and further evaluated its application through a quasi-experimental design. The findings showed that, compared with the control group, the intervention group had a lower incidence of postoperative delirium, lower peak CAM-S scores, and a shorter duration of delirium. The overall incidence of delirium-related adverse events was also reduced in the intervention group. In addition, the intervention group demonstrated better outcomes in quality of life, negative emotional status, selected postoperative recovery indicators, and nursing satisfaction. These findings suggest that the Neuman Systems Model–based prevention and management protocol is feasible and may have meaningful clinical value in the management of postoperative delirium in patients undergoing pancreatic cancer surgery. Postoperative delirium is widely regarded as the result of the combined influence of multiple predisposing and precipitating factors, and multicomponent non-pharmacological interventions are currently among the most strongly supported preventive strategies.^[17-20]^ Accordingly, shifting the management of postoperative delirium in pancreatic cancer from a reactive model focused on treatment after onset to a proactive model emphasizing prevention throughout the perioperative period is supported by both theoretical rationale and clinical relevance.

In the present study, the incidence of postoperative delirium was lower in the intervention group than in the control group, and both delirium severity and duration were correspondingly reduced. These findings are consistent with previous evidence supporting multicomponent interventions for delirium prevention. Inouye et al reported a significant reduction in delirium incidence among hospitalized older adults through a multicomponent intervention strategy. A meta-analysis by Hshieh et al, together with a systematic review of the Hospital Elder Life Program, further demonstrated that structured management targeting modifiable risk factors, including cognitive orientation, sleep maintenance, mobility promotion, sensory support, and prevention of dehydration, can reduce the incidence of delirium and related adverse events.^[18]^ Marcantonio also emphasized that proactive identification and comprehensive management of modifiable risk factors represent one of the most practical approaches to delirium prevention and treatment in hospitalized patients.^[21]^ In addition, a randomized controlled trial by Wang et al showed that individualized Hospital Elder Life Program-based interventions involving family participation could further reduce delirium incidence and improve functional recovery in older postoperative patients.^[22]^ Evidence from pancreatic surgery has likewise suggested that patients undergoing pancreaticoduodenectomy, who often experience substantial surgical trauma and complex perioperative stress, are particularly susceptible to postoperative delirium and therefore may benefit from early identification and continuous management. More recent studies have also proposed that assessment of delirium interventions should extend beyond the simple occurrence of delirium, as severity and duration provide additional insight into the patient’s clinical burden and short-term prognosis.^[23]^ This perspective is consistent with the design of the present study, which simultaneously evaluated delirium incidence, peak CAM-S score, and delirium duration. Taken together, these findings suggest that for multifactorial perioperative complications such as postoperative delirium in patients with pancreatic cancer, a structured, staged, and operational prevention and management pathway may offer greater clinical value than isolated nursing measures.

It is important to recognize that the management of postoperative delirium in patients with pancreatic cancer is highly population-specific. Compared with general surgical patients, this population is often exposed to greater metabolic instability, a more complex postoperative complication profile, and more substantial psychological stress. Following pancreatectomy, patients commonly experience glycemic fluctuation, fluid and electrolyte imbalance, multiple indwelling catheters, increased susceptibility to infection, and restricted nutritional intake. These factors may not only hinder postoperative recovery but also act synergistically to increase the risk of delirium. In addition to well-established risk factors such as advanced age, infection, electrolyte disturbance, and malnutrition, recent studies have suggested that the depth of anesthesia, exposure to sedative and analgesic agents, heightened inflammatory responses, and environmental stimulation in the intensive care unit may further exacerbate neurocognitive vulnerability in high-risk patients.^[24]^ Other evidence indicates that postoperative delirium after complex major surgery is rarely attributable to a single factor; rather, it typically arises from the combined effects of diminished baseline physiological reserve, intensified perioperative stress, and adverse environmental stimuli.^[25]^ Accordingly, postoperative delirium in patients with pancreatic cancer should not be regarded merely as an isolated neuropsychiatric manifestation but rather as a complex management issue shaped by the interaction of physiological, psychological, environmental, and social support–related stressors. The protocol developed in this study was designed to address these population-specific characteristics by integrating preoperative screening, psychological support, perioperative physiological stress management, environmental optimization, and family involvement, thereby better aligning with the practical demands of delirium prevention and management in patients undergoing pancreatic cancer surgery.

From a theoretical perspective, the findings of this study primarily support the feasibility of management strategies guided by the Neuman Systems Model rather than constituting a direct validation of the model itself. The Neuman Systems Model conceptualizes the individual as an open system that maintains dynamic equilibrium under the combined influence of stressors at multiple levels, including physiological, psychological, sociocultural, and environmental domains. Within this framework, the central task of nursing is to identify these stressors and address them through graded preventive interventions. In the setting of postoperative delirium after pancreatic cancer surgery, factors such as pain, infection, sleep disturbance, environmental stimulation, disorientation, and inadequate family support rarely occur in isolation; instead, they are often interrelated and jointly influence postoperative recovery. Skalski et al pointed out that one of the major strengths of the Neuman Systems Model is its capacity to help nurses interpret complex clinical problems through the lens of multidimensional stressors. Similarly, studies by Akhlaghi et al in patients undergoing cardiovascular surgery suggested that interventions developed on the basis of this model can improve perioperative anxiety and stress responses. At the same time, recent research on pathway-based management has indicated that the successful translation of a theoretical model into clinical benefit depends not only on its conceptual explanatory value but also on its ability to support the practical organization of risk assessment, stratified intervention, and team collaboration.^[26]^ Other studies have further shown that combining standardized screening procedures with nursing pathway management can improve the identification of high-risk patients and enhance the consistency of management practices.^[27]^ Taken together with the findings of the present study, these observations suggest that the principal value of the Neuman Systems Model in the management of postoperative delirium in patients with pancreatic cancer lies in its ability to integrate otherwise fragmented nursing measures into a coherent preventive management pathway and to provide an operational framework for addressing complex perioperative nursing challenges.

This study also found that the intervention group had higher quality of life scores at 1 and 3 months after surgery, lower depression, anxiety, and stress scores at postoperative day 7, shorter hospital stays, lower drainage volumes, and more favorable mean blood glucose and albumin levels than the control group. These findings suggest that the benefits of the preventive management protocol may extend beyond reducing delirium episodes alone and may also contribute to improvements in selected perioperative recovery outcomes. Nevertheless, these results should be interpreted with caution. First, a reduction in delirium severity may itself be associated with better short-term recovery; studies using the CAM-S have shown that greater delirium severity is linked to a higher risk of adverse in-hospital outcomes and poorer functional recovery after discharge. Second, the intervention in this study targeted multiple domains, including sleep, nutrition, infection, electrolyte balance, and psychological status, and its effects were therefore not confined to delirium prevention alone. Third, the analyses of quality of life and negative emotions were based on between-group comparisons at individual time points rather than on a repeated-measures model; accordingly, overly strong inferences regarding time effects or interaction effects should be avoided. Previous studies have suggested that perioperative delirium prevention programs may indirectly improve subjective outcomes and recovery experiences by enhancing overall care quality, strengthening orientation support, and promoting family involvement.^[28]^ Other evidence indicates that multicomponent intervention pathways may provide additional benefits in improving postoperative recovery experiences, facilitating early functional recovery, and shortening the recovery process, although the underlying mechanisms remain to be clarified.^[29]^ Therefore, a more cautious interpretation would be that the intervention group performed better on several patient-reported outcomes and postoperative recovery indicators, suggesting that the program may have a positive effect on overall recovery; however, this conclusion requires further confirmation in studies with larger sample sizes and more rigorous statistical designs.

Supplementary analyses in this study showed that age ≥ 70 years, preoperative sleep disturbance, electrolyte imbalance within 7 days after surgery, infection within 7 days after surgery, and preoperative malnutrition were independently associated with postoperative delirium. Among these factors, electrolyte imbalance within 7 days postoperatively and age ≥ 70 years demonstrated relatively stronger associations with delirium. These findings are broadly consistent with previous literature. Inouye et al identified advanced age as one of the most consistent risk factors for delirium, while infection, sleep disturbance, and acute physiological derangements are recognized as common precipitating factors. Gallagher et al and Tomimaru et al similarly reported that age and perioperative complexity were closely associated with postoperative delirium in patients undergoing pancreaticoduodenectomy. Studies by Ito et al and Zhao et al further suggested that advanced age, infection, electrolyte disturbance, and poor baseline nutritional status are associated with the development of postoperative delirium after pancreatic surgery. In addition, studies involving patients undergoing gastrointestinal cancer surgery have shown that malnutrition, sleep disturbance, and perioperative infection are significantly related to postoperative delirium. More recent work on risk prediction and electronic medical record–based stratification has also supported the importance of advanced age, infection, biochemical abnormalities, and nutritional vulnerability in identifying patients at high risk for postoperative delirium.^[30]^ Other studies have indicated that early risk stratification tools may help clinical teams identify vulnerable patients at an earlier stage and thereby improve the precision of preventive management.^[31]^ It should be emphasized, however, that Tables 8 and 9 in the present study were intended primarily to supplement the description of factors associated with postoperative delirium in patients with pancreatic cancer and to support the rationale underlying protocol development, rather than to establish a mature predictive model. In other words, the value of these analyses lies more in explaining why the present protocol specifically targeted sleep, electrolyte balance, infection, and nutrition than in constructing an independent risk prediction system.

This study has several limitations. First, a historical control design was used. Although both study phases were conducted at the same center, within the same specialized ward, and under a comparable perioperative care framework, efforts were made to maintain consistency in routine practices outside the study protocol; however, temporal trend bias and nonrandom allocation bias could not be completely eliminated. Second, this was a single-center study with a relatively small sample size; therefore, the generalizability of the findings requires further confirmation in large-scale multicenter studies. Third, although standardized checklists were used to document protocol implementation, adherence to individual intervention components was not quantified, nor were dose–response relationships between specific components and outcomes examined. In addition, nursing satisfaction is a subjective outcome that may be influenced by patient expectations, communication experience, and individual psychological status; therefore, the related findings should be interpreted cautiously. Moreover, for outcomes assessed at multiple time points, such as quality of life and emotional status, this study used between-group comparisons at each time point rather than a repeated-measures model, which limits the rigor of statistical inference. Future studies should adopt prospective multicenter designs, further refine statistical methods, and incorporate risk stratification tools, process evaluation, and implementation science approaches to better clarify intervention fidelity, the contribution of key components, and applicability across different patient subgroups.

Nevertheless, this study has several potential clinical implications. First, in response to the longstanding lack of a systematic nursing pathway for postoperative delirium in patients with pancreatic cancer, a population at particularly high risk, this study attempted to develop a prevention and management protocol guided by the Neuman Systems Model and to evaluate its preliminary application in a real-world clinical setting. Second, the findings suggest that the management of postoperative delirium in this population should give particular attention to advanced age, sleep disturbance, electrolyte imbalance, infection, and malnutrition, and that these factors should be incorporated into perioperative assessment and ongoing management. In addition, the study highlights that the value of theoretical models in perioperative care may lie less in directly validating the theoretical framework itself than in their ability to help clinical teams organize otherwise fragmented measures into a structured, actionable, and assessable management pathway. Overall, the Neuman Systems Model–based protocol for the prevention and management of postoperative delirium in patients with pancreatic cancer showed favorable trends in reducing delirium incidence, alleviating delirium burden, and improving selected recovery outcomes. However, further validation and refinement through higher-quality studies will still be required.

## Author contributions

**Conceptualization:** Shujia Wang, Li Yu, Wenli Xu.

**Data curation:** Shujia Wang, Li Yu, Wenli Xu.

**Formal analysis:** Shujia Wang, Li Yu, Wenli Xu.

**Funding acquisition:** Wenli Xu.

**Investigation:** Wenli Xu.

**Writing – original draft:** Wenli Xu.

**Writing – review & editing:** Wenli Xu.

## References

[1] GBD 2017 Pancreatic Cancer Collaborators. The global, regional, and national burden of pancreatic cancer and its attributable risk factors in 195 countries and territories, 1990-2017: a systematic analysis for the global burden of disease study 2017. Lancet Gastroenterol Hepatol. 2019;4:934–47. 31648972 10.1016/S2468-1253(19)30347-4 PMC7026711

[2] Siegel RL Miller KD Wagle NS Jemal A. Cancer statistics, 2023. CA Cancer J Clin. 2023;73:17–48. 36633525 10.3322/caac.21763

[3] Klein AP. Pancreatic cancer: a growing burden. Lancet Gastroenterol Hepatol. 2019;4:895–6. 31648975 10.1016/S2468-1253(19)30323-1 PMC7376745

[4] Inouye SK Westendorp RG Saczynski JS. Delirium in elderly people. Lancet. 2014;383:911–22. 23992774 10.1016/S0140-6736(13)60688-1 PMC4120864

[5] Hshieh TT Yue J Oh E. Effectiveness of multicomponent nonpharmacological delirium interventions: a meta-analysis. JAMA Intern Med. 2015;175:512–20. 25643002 10.1001/jamainternmed.2014.7779 PMC4388802

[6] Aldecoa C Bettelli G Bilotta F. European Society of Anaesthesiology evidence-based and consensus-based guideline on postoperative delirium. Eur J Anaesthesiol. 2017;34:192–214. 28187050 10.1097/EJA.0000000000000594

[7] Gallagher TK McErlean S O’Farrell A. Incidence and risk factors of delirium in patients post pancreaticoduodenectomy. HPB (Oxford). 2014;16:864–9. 24750484 10.1111/hpb.12266 PMC4159461

[8] Ito Y Abe Y Handa K. Postoperative Delirium in Patients after Pancreaticoduodenectomy. Dig Surg. 2017;34:78–85. 27463247 10.1159/000446928

[9] Tomimaru Y Park SA Shibata A. Predictive factors of postoperative delirium in patients after pancreaticoduodenectomy. J Gastrointest Surg. 2020;24:849–54. 30941686 10.1007/s11605-019-04212-1

[10] Zhao B Ji HS Xu CY. Incidence and risk factors of postoperative delirium after pancreatic cancer surgery: a retrospective study. Surg Today. 2023;53:736–42. 36335219 10.1007/s00595-022-02614-4

[11] Skalski CA DiGerolamo L Gigliotti E. Stressors in five client populations: neuman systems model-based literature review. J Adv Nurs. 2006;56:69–78. 16972920 10.1111/j.1365-2648.2006.03981.x

[12] Akhlaghi E Babaei S Abolhassani S. Modifying stressors using betty neuman system modeling in coronary artery bypass graft: a randomized clinical trial. J Caring Sci. 2020;9:13–9. Published 2020 Mar 1 32296654 10.34172/jcs.2020.003 PMC7146725

[13] Akhlaghi E Babaei S Mardani A Eskandari F. The effect of the neuman systems model on anxiety in patients undergoing coronary artery bypass graft: a randomized controlled trial. J Nurs Res. 2021;29:e162. Published 2021 Jun 2 34074963 10.1097/JNR.0000000000000436

[14] Inouye SK van Dyck CH Alessi CA Balkin S Siegal AP Horwitz RI. Clarifying confusion: the confusion assessment method. A new method for detection of delirium. Ann Intern Med. 1990;113:941–8. 2240918 10.7326/0003-4819-113-12-941

[15] Inouye SK Kosar CM Tommet D. The CAM-S: development and validation of a new scoring system for delirium severity in 2 cohorts. Ann Intern Med. 2014;160:526–33. 24733193 10.7326/M13-1927 PMC4038434

[16] Sugi T Enomoto T Ohara Y. Risk factors for postoperative delirium in elderly patients undergoing gastroenterological surgery: a single-center retrospective study. Ann Gastroenterol Surg. 2023;7:832–40. Published 2023 Apr 25 37663963 10.1002/ags3.12676 PMC10472384

[17] Inouye SK Bogardus ST Jr Charpentier PA. A multicomponent intervention to prevent delirium in hospitalized older patients. N Engl J Med. 1999;340:669–76. 10053175 10.1056/NEJM199903043400901

[18] Hshieh TT Yang T Gartaganis SL Yue J Inouye SK. Hospital elder life program: systematic review and meta-analysis of effectiveness. Am J Geriatr Psychiatry. 2018;26:1015–33. 30076080 10.1016/j.jagp.2018.06.007 PMC6362826

[19] Oh ES Fong TG Hshieh TT Inouye SK. Delirium in older persons: advances in diagnosis and treatment. JAMA. 2017;318:1161–74. 28973626 10.1001/jama.2017.12067 PMC5717753

[20] Aldecoa C Bettelli G Bilotta F. Update of the European society of anaesthesiology and intensive care medicine evidence-based and consensus-based guideline on postoperative delirium in adult patients. Eur J Anaesthesiol. 2024;41:81–108. 37599617 10.1097/EJA.0000000000001876 PMC10763721

[21] Marcantonio ER. Delirium in hospitalized older adults. N Engl J Med. 2017;377:1456–66. 29020579 10.1056/NEJMcp1605501 PMC5706782

[22] Wang YY Yue JR Xie DM. Effect of the tailored, family-involved hospital elder life program on postoperative delirium and function in older adults: a randomized clinical trial. JAMA Intern Med. 2020;180:17–25. 31633738 10.1001/jamainternmed.2019.4446 PMC6806427

[23] Vasunilashorn SM Fong TG Helfand BKI. Psychometric properties of a delirium severity score for older adults and association with hospital and posthospital outcomes. JAMA Netw Open. 2022;5:e226129. Published 2022 Mar 1 35357447 10.1001/jamanetworkopen.2022.6129 PMC8972033

[24] Wang Y Zhu H Xu F Ding Y Zhao S Chen X. The effect of anesthetic depth on postoperative delirium in older adults: a systematic review and meta-analysis. BMC Geriatr. 2023;23:719. Published 2023 Nov 6 37932677 10.1186/s12877-023-04432-w PMC10629190

[25] Hoogma DF Milisen K Rex S Tmimi L A. Postoperative delirium: identifying the patient at risk and altering the course: a narrative review. Eur J Anaesthesiol Intensive Care. 2023;2:e0022. Published 2023 Apr 26 39917289 10.1097/EA9.0000000000000022 PMC11783674

[26] Shen H Liu X Wu L Jia J Jin X. Effect of hospital elder life program on the incidence of delirium: A systematic review and meta-analysis of clinical trials. Geriatr Nurs. 2024;56:225–36. 38367545 10.1016/j.gerinurse.2024.02.017

[27] Burton JK Craig LE Yong SQ. Non-pharmacological interventions for preventing delirium in hospitalised non-ICU patients. Cochrane Database Syst Rev. 2021;7:CD013307. Published 2021 Jul 19 34280303 10.1002/14651858.CD013307.pub2 PMC8407051

[28] Li J Fan Y Luo R. Family involvement in preventing delirium in critically ill patients: a systematic review and meta-analysis. Int J Nurs Stud. 2025;161:104937. 39486106 10.1016/j.ijnurstu.2024.104937

[29] Chen CC Li HC Liang JT. Effect of a modified hospital elder life program on delirium and length of hospital stay in patients undergoing abdominal surgery: a cluster randomized clinical trial. JAMA Surg. 2017;152:827–34. 28538964 10.1001/jamasurg.2017.1083 PMC5710459

[30] Rieck KM Pagali S Miller DM. Delirium in hospitalized older adults. Hosp Pract (1995). 2020;48:3–16. 31874064 10.1080/21548331.2019.1709359

[31] Lindroth H Bratzke L Purvis S. Systematic review of prediction models for delirium in the older adult inpatient. BMJ Open. 2018;8:e019223. Published 2018 Apr 28 10.1136/bmjopen-2017-019223 PMC5931306 29705752

